# Exploring Barriers to Healthy Eating Among Women in Their Role as New Mothers with a Theory-Driven Questionnaire

**DOI:** 10.1007/s10995-023-03622-7

**Published:** 2023-04-03

**Authors:** Andreia Ferreira Moura, Jessica Aschemann-Witzel

**Affiliations:** 1 Department of Food, Nutrition and Dietetics. School of Biosciences, University of Nottingham, Sutton Bonington, Loughborough, LE12 5RD UK; 2 Department of Management, MAPP Centre for Research On Value Creation in the Food Sector, BSS, Aarhus University, Fuglesangsalle 4, 8210 Aarhus V, Denmark

**Keywords:** Mothers, Healthy eating, Denmark, Social Cognitive Theory

## Abstract

**Objectives:**

This study aims to propose and evaluate a theory-driven questionnaire addressing barriers to healthy eating among mothers of young children.

**Methods:**

Statements drawing upon the Social Cognitive Theory were developed/gathered based on literature review and previous qualitative research. Part I (43 items) included general barriers, attitudes to nutrition advice and outcome expectations. Part II (9 items) included subjective knowledge and general self-efficacy scales. An online survey was undertaken with 267 Danish women. The validation process included content and face validity, exploratory factor analysis (EFA) and reliability analysis. Confirmatory factor analysis (CFA) tested possible associations between the constructs and potential health outcomes (BMI and healthiness of eating habits).

**Results:**

The EFA supported an adequate factorial validity with a 5-factor, 37-item structure model for Part I, and a high internal reliability of Parts I and II (Cronbach’s alpha > 0.7). The CFA revealed an association between certain constructs and perceived healthiness of eating and BMI. Results support the reliability and factorial validity of the social cognitive measures assessing barriers to healthy eating among mothers.

**Conclusions for Practice:**

These promising findings of reliability and initial validity suggest that researchers and practitioners interested in identifying women who face difficulties in the family food environment may find the scales useful. We propose a short version of the questionnaire for health practitioners.

**Supplementary Information:**

The online version contains supplementary material available at 10.1007/s10995-023-03622-7.

## Significance

*What is already known on this subject?* Women have reported a lack of attention to their needs by health practitioners during the transition to parenthood. Health professionals explain that there is a certain negligence of parental dietary health in antenatal and postnatal care due to a lack of time and resources for nutrition counseling.

*What does this study add?* We provide a potentially reliable theory-based tool for assessing constraints to healthy eating among mothers of young children. The study answers a call for evidence-based instruments to support that the work of midwives and other practitioners involved in new mothers’ and their families’ care is time-optimized.

**Supplementary Information:**

The online version contains supplementary material available at 10.1007/s10995-023-03622-7.

## Introduction

Unhealthy dietary patterns account for a large proportion of the greatest health and environmental challenges of the twenty-first century, including obesity, non-communicable diseases and environmental degradation (Willett et al., 2019). The negative impact of unhealthy eating affects all countries (Hawkes & Popkin, 2015), and this calls for action. However, healthy eating in the existing food system is complex. Many personal, social and environmental obstacles act against individuals’ intentions to eat healthy foods. These barriers seem to affect mothers of young children in particular, due to the strains of childbearing and the obligations of family life. For instance, Danish women reported a perceived downturn in healthy eating after becoming mothers, mainly due to time constraints associated with a busy and stressful routine (Moura & Aschemann-Witzel, 2020). Indeed, the transition to parenthood is reported to challenge women’s healthy dietary patterns (Nasuti et al., 2014).

The investigation of the barriers to healthy eating among mothers is of great importance because parents shape their children’s early learning about food and eating, thus influencing the children’s dietary behaviors and health for life (Birch & Ventura, 2009). Moreover, understanding the constraints for a healthy diet in the family food environment might provide insights of value for developing childhood obesity prevention programs (Luecking et al., 2017). A problem-centered investigation targeting women in their role as mothers might enable us to identify key triggers of unhealthful food practices and possible points of intervention, and at the same time ensure that women’s needs and wants remain a central focus.

Such an approach seems particularly important for healthcare providers involved during the transition to parenthood. Previous studies have reported a perceived lack of attention to parents’ needs by health practitioners (Christenson et al., 2016; Moura & Aschemann-Witzel, 2020). Women in several countries reported that they received very little dietary information from their midwives (Bloomingdale et al., 2010; Wennberg et al., 2013; Moura & Aschemann-Witzel, 2020). This lack of information is worrying since early parenthood is an opportunity for nutrition intervention. Health services in this period have significant contact with mothers-to-be, and the latter often have an increased interest in health-promoting actions (Bassett-Gunter et al., 2013).

Health professionals explain that there is negligence of parental dietary patterns in antenatal and postnatal care due to a lack of time and resources for nutrition consultations (Bahri Khomami et al., 2021; Lucas et al., 2020). Therefore, it is important to provide healthcare staff with tools that optimize the consultation time (e.g., pre-consultation screening questionnaires). The present research answers a call for evidence-based instruments to support the work of midwives and other health practitioners. These instruments can contribute to the work of those involved in new mothers’ and their families’ care (Bahri Khomami et al., 2021; Bick, 2009).

**Fig. 1:**
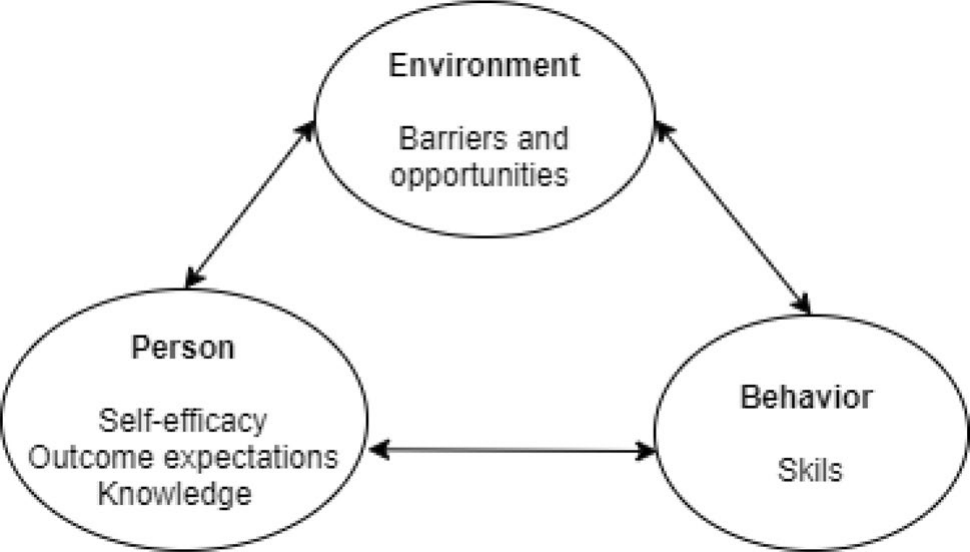
Social Cognitive Theoretical Framework. Based on Kelder et al., (2015)

Most compelling evidence indicates that to have more effective nutrition counseling, theory-based insights should be gathered on the perceived barriers to healthier diets on the one hand, and on the personal benefits expected in relation to eating healthily on the other hand (Luecking et al., 2017). In this regard, the theoretical framework proposed by the Social Cognitive Theory (SCT) is considered a suitable tool, as it contemplates personal, social and environmental barriers to behavior change, and outcome expectations of health behaviors adoption. The central proposal of the theory is a triple causality between behavior, environment and personal factors; which interact to determine each other (Fig. 1) (Bandura, 2004).

Major constructs of the SCT, namely self-efficacy and outcome expectations, provide a comprehensive model for studying parental eating patterns and the ability to create optimal family food environments (Byrd‐Bredbenner et al., 2011). For instance, mothers of young children who declared confidence in their ability to eat healthily (self-efficacy) and who believed in the link between diet and health (outcome expectations), had healthier BMIs and healthier dietary intakes (Byrd‐Bredbenner et al., 2011). In addition, confidence in cooking skills (also related to self-efficacy) is linked to more frequent meal planning, purchase of more fresh foods (Reicks et al., 2014) and consequently healthier meals in the household (Beshara et al., 2010).

Self-efficacy is considered the central component of the SCT in relation to nutrition behavior (Anderson et al., 2000). Self-efficacy is an essential point to explore among new parents, considering that general self-efficacy might decrease in the transition to parenthood, as a consequence of a decreased sense of freedom and power to achieve personal goals with the arrival of a child (Nomaguchi & Milkie, 2003). However, self-efficacy is only relevant when individuals expect to obtain positive outcomes from a certain behavior. For example, higher cooking self-efficacy combined with positive outcome expectations (e.g., an enjoyable meal) may increase the motivation for, or the intention to engage in, meal preparation despite obstacles (Beshara et al., 2010).

In light of this, the present study aims to develop and evaluate a theory-driven survey instrument addressing social-cognitive factors for healthy eating in the family food environment targeted at mothers of young children. The final goal is to propose a tool that is suitable for health practitioners’ use. An informative questionnaire that can be answered before the consultation might help practitioners who lack sufficient time to engage in more client-centered dialogues.

## Methods

### Questionnaire Development

The study presented here was part of a larger cross-country project investigating barriers to healthy eating among parents, building upon previously conducted qualitative research ( Moura & Aschemann-Witzel, 2020). As a theoretical framework, we employed the Social Cognitive Theory, and included statements based on empirical research on the following: (1) constraints to healthy eating in the family food environment, (2) factors associated with food provisioning practices (e.g., “On busy nights, our family’s main meal includes canned or frozen entrees, boxed mixes or microwaveable dinners”, “What we are going to have for dinner is very often a last-minute decision.”), (3) attitudes towards nutrition advice and (4) outcome expectations of healthy eating. Additionally, pre-established scales were included for other important constructs of the SCT: general self-efficacy and (subjective) knowledge. Details on the questionnaire development, references for each item/scale and translation process are included as Online Resource.

The initial questionnaire consisted of 52 statements divided into two parts. Part I (43 items) included (personal, behavioral and environmental) barriers to healthy eating, attitudes toward nutrition advice and positive outcome expectations. In this part, the items were mostly phrased in a negative form (e.g., “I don’t have enough money to buy fruit and/or vegetables”, “I don’t have enough time to prepare healthy food.”). Responses ranged in 7-point Likert-scales of 1 (strongly disagree) to 7 (strongly agree). Thus, higher scores indicated more barriers to healthy eating. Only the scale for “outcome expectations” was phrased positively (e.g., “It’s important that the food I eat helps to prevent diseases”) and ranged from 1 (Very true of me) to 7 (Very untrue of me). Therefore, high scores indicated lower agreement with the positive outcome expectations of healthy eating (so a higher final score in the questionnaire indicates more challenges for health professionals to address).

In Part II (9 items), the subjective knowledge scale was also mostly negatively phrased (e.g., “When it comes to healthy eating, I really don’t know a lot.”). The positively phrased items were reversed so higher scores indicated lower subjective knowledge. Only self-efficacy was phrased positively (e.g., “If I am in a challenging situation, I tend to find a way out”), therefore higher scores indicated higher capabilities in this domain.

### Socio-demographics and (Perceived) Health Measurements

In addition to the scales described above, the survey included personal and socio-demographic questions (age, weight, height, household structure, education, etc.) and a question about the perceived healthiness of eating in distinct phases of becoming a mother: pregnancy, first weeks with the baby and nowadays (Moura & Aschemann-Witzel, 2020). The questions were phrased as follows: “To which extent do you think that you ate more/less healthy during pregnancy, compared to before being pregnant?”, “To which extent do you think that you ate more/less healthy during the first weeks of the baby, compared to before having a child?” Next: “Please think about your current eating habits. To which extent do you think that you eat more/ less healthy now, compared to before being a mother?” The alternatives ranged from “1: Much less healthy” to “7: Much healthier”. With those questions, we aimed to determine whether the social-cognitive barriers to healthy eating might influence the impact of motherhood on eating behaviors*.*

### Sample

The study was restricted to women, acknowledging that women in their role as mothers are the primary target of nutrition advice from health professionals in the transition to parenthood (Edvardsson et al., 2011) and might give more insights into attitudes to nutrition counseling. Furthermore, women remain primarily responsible for household work and food-related tasks, enabling them more accurately to point out the barriers to healthy eating (Kan et al., 2011).

Women living in Denmark who had at least one child up to the age of 4 years were eligible to participate. This preschool age was observed to be meaningful in regard to perceived changes in healthy eating behaviors related to motherhood (Moura & Aschemann-Witzel, 2020). For the recruitment of survey sample participants, we contacted Userneeds, a Danish research company (https://userneeds.com/en/). The company holds an online consumer panel representative for the Danish population. Userneeds’ panel members received invitations to participate in an online survey that would help researchers “to investigate the challenges and opportunities related to food consumption among mothers of young children”. Quotas applied to the completed survey responses ensured that the sample represented the main regions in the country. The study was approved by Aarhus University’s ethics committee (serial number: 2019-28), which follows the ethical standards laid down in the 1964 Declaration of Helsinki and its later amendments. All respondents gave their informed consent prior to their inclusion in the study.

Three hundred women completed the survey, but 33 of them were excluded because they completed the survey in less than five minutes (considered not to be sufficient to read and answer all questions attentively and properly). The final sample thus included 267 women. The sample size was acceptable considering the internal consistency and factor analysis criteria. We considered the minimum internal consistency to be set at 0.5, and the first (largest) eigen values from Exploratory Factor Analysis (EFA) to be between 3.00 and 6.00, which was reported to be achieved with a minimum sample size of 100 participants (Yurdugül, 2008). Further details on the EFA are available as Online Resource.

The women were on average 32 years old (± 5.4) with a slightly high weight and Body Mass Index (mean BMI 26 (± 9.3), calculated based on self-reported weight and height). Most had one or two children who were 4 years old or less. The majority were well educated (2 years of university degree or more), living with a spouse/partner (90%) and working full time (53%) (Table 1). Compared to the population of the country, the final sample is representative of women’s educational status in Denmark (OECD, 2019). The mean age in the sample (32 years) is approximately comparable to the mean age of first-time mothers in the country (29 years) (Statistics Denmark, 2020).

**Table 1:** Mothers’ Socio-demographic Characteristics

*N*: 267	Mean (SD)
Age	32 (5,4)
BMI	26 (9,3)

BMI	n	%
Less than 18.5	7	3
18.5 to 24.9	126	47
25 to 29.9	65	24
30 or more	39	15
*“I do not want to answer”*	30	11
Number of children	n	%
Mothers with 1 child only	119	45
Mothers with 2 children	117	44
Mothers with 3 children	26	9,6
Mothers with 4 children	4	1
Mothers with 5 children	1	0,4
Age of children		
*Youngest child*	n	%
Mothers whose youngest child was *younger* than 12 months	58	22
Mothers whose youngest child was *older* than 12 months (between 12 and 48 months old)	209	78
*Oldest child*	n	%
Mothers whose oldest (or only) child was *younger than 12 months*	26	10
Mothers whose oldest child was between *12 and 48 months*	162	60
Mothers whose oldest child was between *5 and 8 years old*	52	19
Mothers whose oldest child was *older than 8 years old*	27	11
Pregnancy		
Mothers who declared to be pregnant at the moment of the survey	26	10
Breastfeeding	n	%
Mothers who declared to be pregnant at the moment of the survey	58	22
Dietary restriction (vegetarian, vegan, gluten/lactose free, other)	51	20
Household structure		
Living with a partner	238	90
Share of food-related activities^a^		
Mother herself	120	45
Education	n	%
1 = Lower secondary education or less	8	3
2 = Upper secondary education	23	9
3 = Vocational education	50	19
4 = Short higher education (up to 2 years)	28	10
5 = Higher education (2–4 years including bachelor’s degree)	100	37
6 = More than 4 years of higher education (including a master’s degree	53	20
7 = Ph.D	5	2
Employment status		
Working full-time (37 or more h/week)	141	53
Perception of financial situation		
Scale from 1 = Difficult 7 = Well off		
1–2	20	7,5
3–5	135	50.5
6 or more	102	38
*“I do not want to answer”*	10	4

^a^Mothers were asked: “If you live with a partner, please indicate who is the main responsible for food-related activities (planning meals, cooking, etc.) in your house”

### Data Analysis

**Fig. 2:**
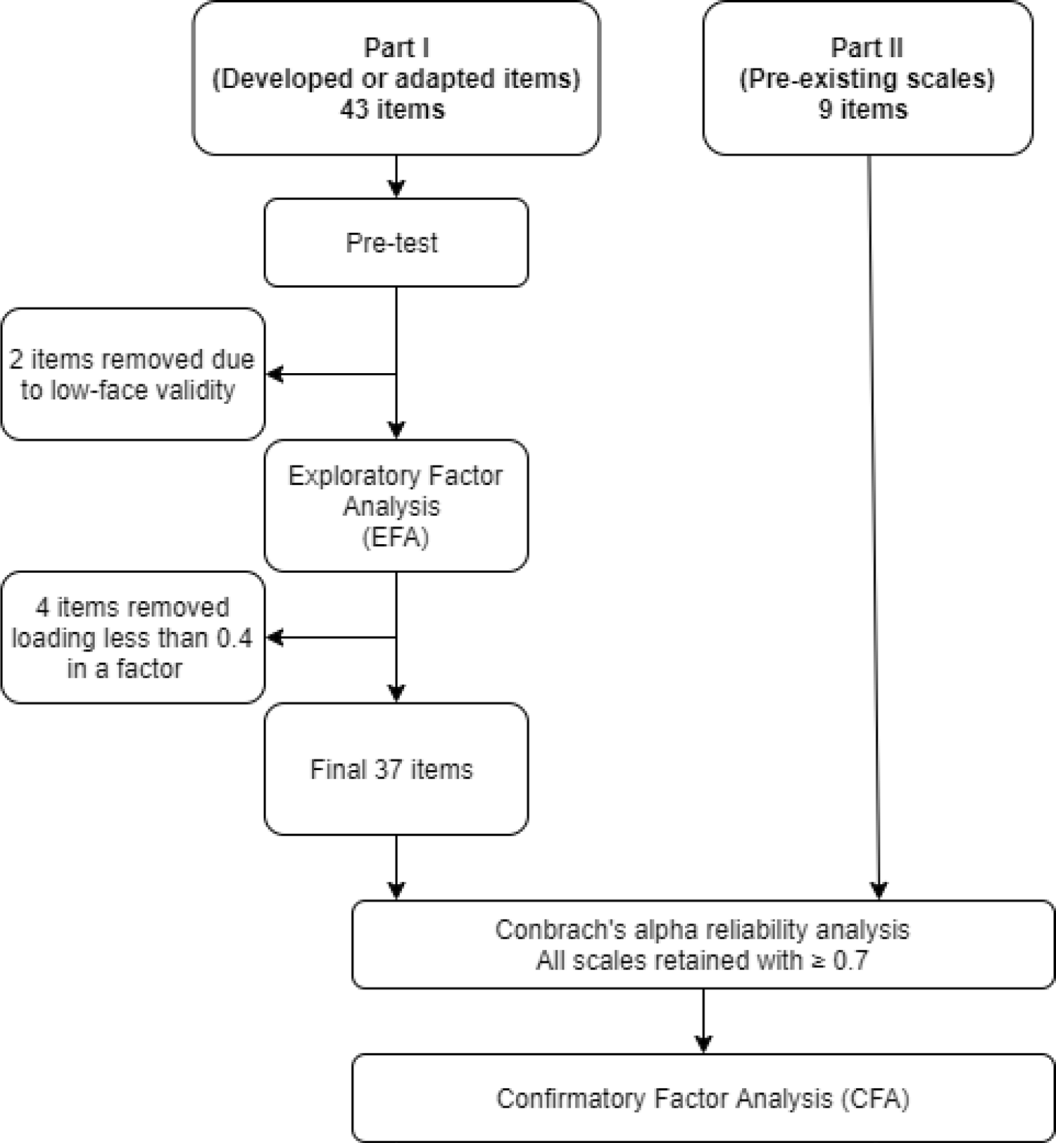
Data analysis structure. *Source*: Own

The data analysis is summarized in Fig. 2 and explained below.

Exploratory Factor analysis (EFA) with oblimin rotation was conducted for Part I of the questionnaire (developed or adapted items). Items with a factor loading of less than 0.4 were removed from the questionnaire (DeVon et al., 2007). Part II (pre-existing scales on social-cognitive measurements) was not included in the factor analysis, since the scales have been validated in previous studies (Flynn & Goldsmith, 1999; Schwarzer, 1993). Cronbach's alpha value was calculated for each of those scales and each construct factor. We retained factors that showed sufficient reliability (Cronbach alpha ≥ 0.7) and had at least three items. Data were analyzed using the Statistical Package for Social Sciences (SPSS version 26.0 for Windows, 2019, SPSS Inc., Chicago).

Confirmatory Factor Analysis (CFA) was conducted using Analysis of Moment Structure (AMOS version 27.0 for Windows, 2019, IBM, Chicago) to evaluate the measurement model fit of the scales, and to confirm the potential relations of the items with certain outcomes (perceived healthiness of eating along the phases of becoming a mother and the BMI). Further details on the statistical validation are included as Online Resource.

## Results

### Exploratory Factor Analysis and Item Optimization

The EFA of Part I of the questionnaire revealed a 5-factor, 37-item structure model: Factor 1: General barriers to healthy eating (22 items), Factor 2: Planning meals (3 items), Factor 3: Cooking enjoyment (3 items), Factor 4: Attitudes towards nutrition advice (4 items), Factor 5: Outcome expectations (5 items). The item loadings are presented in Table 2. Items that loaded onto two separate constructs were retained if they loaded in one factor more than 0.3 from the other (Lanario et al., 2020).

**Table 2:** Factor structure of the questionnaire (Part I—developed or adapted items)

	Loadings	Factor 1	Factor 2	Factor 3	Factor 4	Factor 5
	Mean (SD)	Barriers	Planning	Cooking	Nutr. advice	Outcome expect
*Factor 1: General barriers to healthy eating*^a^						
1.The price of healthy foods is too high	4.60 (1.54)	.439				
2. I do not have enough money to buy fruit and/or vegetables	2.39 (1.37)	.668				
3. I do not have enough money to eat healthily	2.45 (1.37)	.687				
4. It’s difficult to find fresh fruit and vegetables where I live	2.28 (1.34)	.522				
5. Most fresh fruit and vegetables do not look appealing in the store	3.20 (1.58)	.557				
6. I live far from supermarkets or vegetable stores with fresh foods	2.14 (1.35)	.539				
7.It is difficult to find ways to prepare vegetables	3.54 (1.66)	.594				
8. I often lack the inspiration to cook healthy dishes	4.57 (1.64)	.528				
9. I lack cooking skills to prepare healthy food	2.67 (1.42)	.657				
10. I don’t have enough time to prepare healthy food	3.55 (1.60)	.569				
11. On busy nights, our family’s main meal includes canned or frozen entrees, boxed mixes or microwaveable dinners	2.73 (1.63)	.537				
12. I often have to abandon cooking plans because of unexpected events (e.g. work demands, kid’s sickness or mood swings)	3.00 (1.55)	.648				
13. At the end of the day I just don't have the energy to whip up a healthy meal	3.51 (1.56)	.759				
14. Healthy foods don’t taste as good as unhealthy foods	2.63 (1.52)	.653				
15. Healthy foods taste bad	1.99 (1.25)	.618				
16. Healthy meals are boring	2.36 (1.48)	.691				
17. I wouldn’t try new healthy foods that I am not used to	2.54 (1.49)	.547				
18. I find it difficult to balance nutrition concerns and my kid’s food preferences	4.02 (1.59)	.460		.408		
19. I get tired of putting effort into trying new dishes that my family refuses to eat	3.72 (1.67)	.531		.409		
20. My family wastes too much food when I serve fruit and vegetables	3.04 (1.60)	.710				
21. If I were to add more vegetables to my usual dishes, no one in my family would eat them	2.95 (1.57)	.640				
22. If I were to serve fruit for dessert, no one in my family would eat it	2.21 (1.45)	.594				
*Factor 2: Planning meals*^a^						
1. I often ‘‘go with the flow’’ and do not plan meals	3.85 (1.76)	.411	.743			
2. What we are going to have for dinner is very often a last-minute decision	3.53 (1.75)	.490	.690			
3. Usually I do not decide what to buy until I am in the store	3.07 (1.67)	.481	.657			
*Factor 3. Cooking enjoyment*^a^						
1. I don't like spending too much time on cooking	4.30 (1.68)	.447		.536		
2. Preferably, I spend as little time as possible cooking	4.56 (1.58)	.491		.515		
3. Cooking is a task that is best over and done with	4.03 (1.70)	.465		.474		
*Factor 4. Attitudes towards nutrition advice*^a^						
1. Nutrition advice from professionals is confusing	4.03 (1.56)				.489	
2. I am fed up with all the controversy about healthy eating among experts	4.64 (1.54)				.531	
3. I prefer to rely on my own common sense for healthy eating, rather than listening to experts	5.01 (1.29)				.612	
4. I don’t like to be told by health professionals what I should eat. I can decide on my own	4.34 (1.35)				.634	
*Factor 5. Outcome expectations*^b^						
*It’s important to me that the food I eat…*						
1. …helps to prevent diseases	2.89 (1.22)					.752
2. …is good for my appearance (e.g. skin, hair, nails, teeth)	3.11 (1.31)					.729
3.…helps me to live longer	2.65 (1.20)					.711
4…helps to control my weight	2.38 (1.12)					.485
5… does not compromise the environment	3.06 (1.40)					.519
% variance explained		25%	4.8%	5.7%	5.4%	8%
Cronbach’s alpha		.921	.880	.908	.775	.806
Mean		2.9	3.5	4.3	4.5	2.8

^a^Values ranged from 1 (Strongly disagree) to 7 (Strongly agree)

^b^Values ranged from 1 (Very true of me) to 7 (Very untrue of me)

Those five most statistically and conceptually suitable factors accounted for 49% of the variability in the 37 items. The internal consistencies for all factors exceeded 0.7 suggesting adequate internal reliability (see Table 2). A reliability analysis of Part II of the questionnaire (pre-existing scales on social-cognitive measures) revealed Cronbach’s alpha values > 0.8.

### Confirmatory Factor Analysis

The CFA structural relations gave an acceptable representation of the data and association of the scales with two health outcomes (the perceived healthiness of eating nowadays and the BMI). Standardized path coefficients showed that general barriers accounted moderately for the variance in perceived healthiness of eating. The more barriers to healthy eating, the less healthy women perceive eating nowadays compared to before motherhood (negative correlation).

The results considering the BMI as an outcome indicated various variables of influence. Four constructs (general self-efficacy, attitudes to nutrition advice, outcome expectations and subjective knowledge) accounted for the explained variance in BMI. Specifically, the lower the self-efficacy, the more negative the attitudes were toward nutrition advice. The fewer outcome expectations and the lower scores in subjective knowledge, the more tendency to a higher BMI. Value indices and measurement models of the analysis are available as Online Resource.

### Proposal of a Short Version for Health Professionals

The final goal of the study was to develop a tool for health practitioners working with mothers of young children. To achieve this purpose, we reduced the number of items, keeping only the item that would affect Cronbach’s alpha value the least in case the item was deleted. This procedure was applied for all except for the scale “Outcome expectations” where we kept two items, as Cronbach’s alpha value would be affected equally if any of the items were deleted.

The proposed version is presented in Table 3. A final version with a more coherent ordering of the items for hands-on application is proposed in the appendices (Appendix 1). To increase the practical usefulness of the questionnaire, we added general guidance for health professionals on how to advise women in case of high barriers to healthy eating with suggestions for interventions (Appendix 2). Inspired by the work of Widen and Siega-Riz (2010) and from our prior studies with mothers (Moura & Aschemann-Witzel 2020, Moura & Aschemann-Witzel 2021) we suggest, for example, alternatives on how to deal with the (potential) high costs of healthy eating, how to optimize food-related planning and cooking time, and how to avoid food waste. Interventions involving culinary sessions seem important to enhance food-related skills that can decrease barriers to healthy eating (Harmon et al., 2015; Hollywood et al., 2018).

**Table 3:** Proposal of a Survey Instrument for Health Professionals: Social-cognitive Measures of Maternal Barriers to Healthy Eating

	Factor loadings	Scale’s Cronbach’s alpha	Cronbach's Alpha if item deleted
*Factor 1. General barriers to healthy eating*		.920	
(1) I do not have enough money to eat healthily	.687		.915
(2) Most fresh fruit and vegetables do not look appealing in the store	.557		.917
(3) I lack cooking skills to prepare healthy food	.657		.916
(4) At the end of the day I just don't have the energy to whip up a healthy meal	.759		.914
(5) Healthy meals are boring	.691		.915
(6) My family wastes too much food when I serve fruit and vegetables	.710		.914
*Factor 2. Planning meals*		.880	
(7) What we are going to have for dinner is very often a last-minute decision	.690		.810
*Factor 3. Interest/enjoyment in cooking*		.908	
(8) Preferably, I spend as little time as possible cooking	.515		.849
*Factor 4. Attitudes toward nutrition advice*		.775	
(9) I am fed up with all the controversy about healthy eating among experts	.531		.686
*Factor 5. Outcome expectations*		.806	
It’s important to me that the food I eat…			
(10) a …helps to prevent diseases	.752		.738
(10) b …is good for my appearance (e.g. skin, hair, nails, teeth)	.729		.738
Pre-existing scales			
* General self-efficacy*	.882	.891	
(11) If I am in a challenging situation, I tend to find a way out			.853
* Subjective knowledge*		.829	
(12) Compared to most other people, I know less about healthy eating	.872		.760

### Barriers to Healthy Eating Among the Women in the Study

Although the proposal of a research and practice tool was the main goal of the study, it is interesting to point out which barriers to healthy eating were most frequently reported by the participants. Table 4 presents the scores of the items selected to be part of the version for health professionals (Appendix 1). Overall, the main barriers to healthy eating were related to (preferably) spending as little time as possible on cooking (item 3) and being ‘fed up’ with nutrition controversies (item 10). As for attitudes, capabilities and knowledge, the mean scores demonstrated overall high self-efficacy (mean score above 5 on the item, “If I am in a challenging situation, I tend find a way out”) and high outcome expectations of healthy eating (for example, scores close to 1 = very true of me, on the item “It’s important to me that the food I eat helps to prevent diseases”).

**Table 4:** Socio-cognitive barriers to healthy eating among the mothers of the study

	Mean (SD)
*Part I: General barriers to healthy eating*	
(1) I do not have enough money to eat healthily^a^	2.45 (1.37)
(2) Most fresh fruit and vegetables do not look appealing in the store^a^	3.20 (1.58)
(3) Preferably, I spend as little time as possible on cooking^a^	4.56 (1.58)
(4) I lack cooking skills to prepare healthy food^a^	2.67 (1.42)
(5) What we are going to have for dinner is very often a last-minute decision^a^	3.53 (1.75)
(6) At the end of the day I just don't have the energy to whip up a healthy meal^a^	3.51 (1.56)
(7) My family wastes too much food when I serve fruit and vegetables^a^	3.04 (1.60)
(8) Healthy meals are boring^a^	2.36 (1.48)
*Part II. Attitudes, capabilities, knowledge*	
(9) Compared to most other people, I know less about healthy eating^a^	3.14 (1.24)
(10) I am fed up with all the controversy about healthy eating among experts^a^	4.64 (1.54)
(11) If I am in a challenging situation, I tend to find a way out^a^	5.61 (.99)
(12) It’s important to me that the food I eat…^**b**^	
(a) …helps to prevent diseases^**b**^	2.89 (1.22)
(b) …is good for my appearance (e.g. skin, hair, nails, teeth)^**b**^	3.11 (1.31)

^a^Values ranged from 1 (Strongly disagree) to 7 (Strongly agree)

^b^Values ranged from 1 (Very true of me) to 7 (Very untrue of me)

## Discussion

This article reports the development and evaluation of social-cognitive scales to identify barriers to healthy eating among mothers of young children (≤ 4 years old) living in Denmark. This process resulted in a measure addressing general barriers to healthy eating, meal planning, cooking enjoyment, attitudes towards nutrition advice, outcome expectations, general self-efficacy and subjective knowledge.

Overall, the scales’ structure allows the identification of barriers at the financial, situational, attitudinal and cognitive levels, and covers several steps of the food provision process (planning, purchasing and cooking). We hope that this structure is useful for practitioners to optimize their time and to facilitate a tailored nutrition treatment for women, focusing on overcoming the identified constraints. Suggestions for patient/client guidance and interventions for each barrier are provided in “Appendix 2” and focus mostly on ways to optimize the time and resources for healthy eating and making healthy foods and meals more attractive to the family. In terms of intervention, culinary sessions seem important to facilitate those aspects. Also, interventions focusing on culinary aspects of food and less on nutrition concepts might be better received by individuals who score high on the domain of ‘controversy among nutrition experts’ (thus, demonstrating negative attitudes towards controversies in nutrition advice) (Poulain, 2009).

To the best of our knowledge, this is the first study to propose a tool for health professionals working with mothers of young children, helping them to explore constraints and motivations for healthy eating in the family food environment. Most of the existing questionnaires targeting mothers focus on child-feeding practices and do not include dimensions of maternal eating behaviors. Scales addressing difficulties in healthy eating have mostly focused on weight loss (Welsh et al., 2012) or targeted the general population (López-Azpiazu et al., 1999). Another novelty of the current approach lies in unfolding social-cognitive factors in the family food environment that can potentially be used to identify women at high risk of unhealthy eating. In addition, through an empirical analysis (CFA) we could show that four of the identified factors were significantly associated with important health outcomes (general barriers, outcome expectations, attitudes to nutrition advice and subjective knowledge) among the Danish women. General barriers were associated with lower perceived healthiness of eating nowadays (compared to before being a mother), whereas (low) positive outcome expectations, (negative) attitudes to nutrition advice and (low) subjective knowledge were associated with a higher BMI. Thus, the scales demonstrated to hold the potential to predict healthy eating outcomes and should be explored in larger samples. Although the identified associations of the socio-cognitive factors with BMI must be further explored with more rigorous methods (e.g., with actual BMI measurements, instead of self-reported weight and height), the findings presented here indicate that knowledge, attitudes and expectations might be important aspects to consider when addressing new mothers’ nutrition status.

Although we did not employ objective measures, it seems reasonable to postulate that self-reported barriers to healthy eating among Danish mothers of young children decrease the perceived dietary healthiness. The same applies to outcome expectations, as positive outcome expectations of healthy eating can act as incentives for healthier dietary choices (Bandura, 2004). Our findings are in line with previous analyses identifying a higher BMI among mothers with low scores on positive outcome expectations of healthy eating (Byrd‐Bredbenner et al., 2011). In regard to subjective knowledge, previous research identified that individuals with high scores in this construct are, indeed, more likely to make healthier food choices (Chen, 2016). In the context here, subjective knowledge refers to what a person thinks they know about healthy eating. This concept differs from objective knowledge, defined as what a person actually knows about healthy eating. Regarding eating behaviors, subjective knowledge has great importance when considering that more than the actual information, how the information is perceived is what affects people’s behavior the most (House et al., 2004). The same rationale applies to the concept of self-efficacy, in which the beliefs about one's capabilities have a crucial role in a person’s ability to exert a desired behavior.

**Fig. 3:**
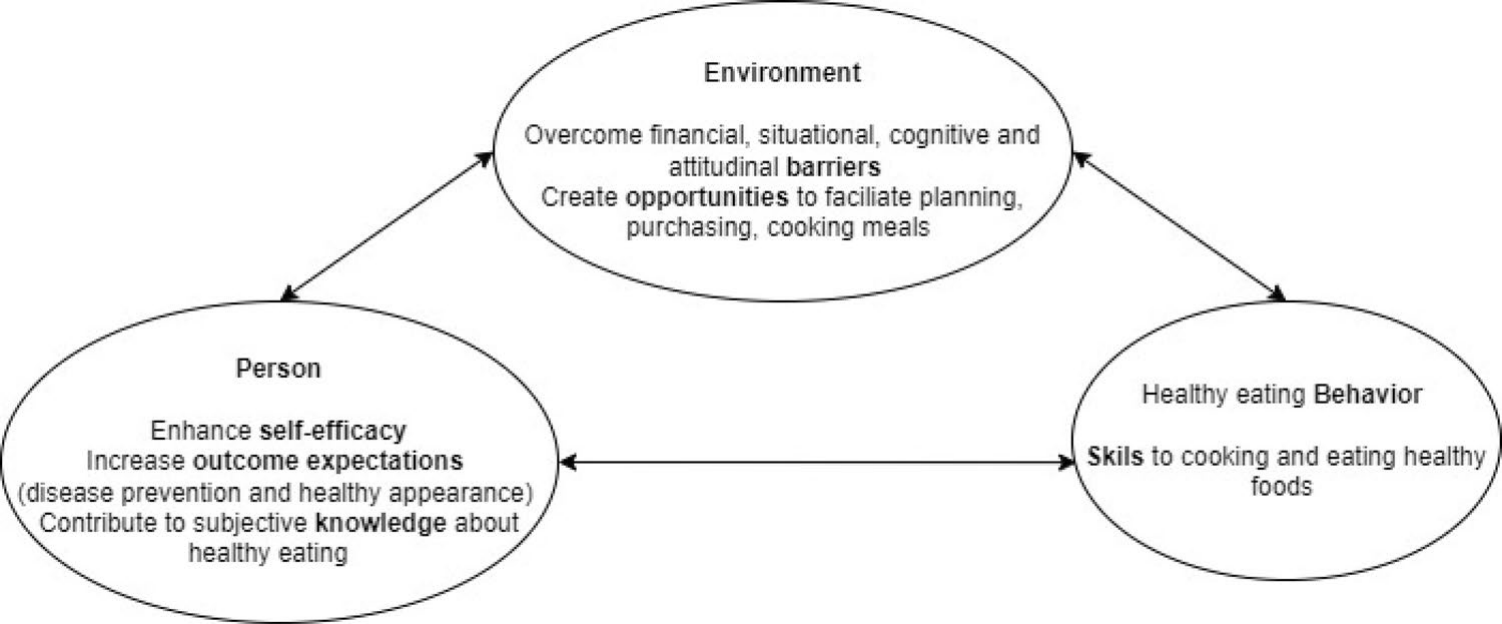
Domains of the Social Cognitive Theoretical framework aligned with the constructs considered in the study. The phrasing refers to guidance for health professionals. The lines connect hypothesized ideas. The length of the lines does not represent importance

Self-efficacy and the other domains of the Social Cognitive Theory were found to be appropriate to investigate barriers to healthy eating among mothers. For example, the fact that the constructs of (low) self-efficacy, (low) outcome expectations and (low) subjective knowledge were associated with a higher BMI, suggests that these aspects explain (at least partially) eating behaviors among the Danish women. The environmental construct ‘general barriers’ was also related to perceived healthy eating (the more the barriers, the lower the perceived healthiness), thus aligning with the framework of the SCT. Although a more robust methodology is needed to confirm these associations, Exploratory Factor Analysis is a recognized technique for exploratory theory generation and validation (Haig, 2005), and allows inferences about theoretical constructs. Drawing upon this technique, Fig. 3 presents the SCT domains aligned with the findings of the study, considering aspects that health professionals can target when working with mothers of young children. In the present study with Danish women, barriers that could be addressed lie in the situational (time to spend cooking) and attitudinal (attitudes to nutrition advice) domains. Other relevant barriers that showed high internal consistency in the questionnaire and that might apply to other women, were financial and cognitive constraints (e.g., the ability to plan meals). Aspects at the individual level that could be addressed to improve healthy eating include self-efficacy, knowledge and outcome expectations (e.g., professionals could consider informing women about the importance of healthy eating for health benefits and good appearance).

The findings that mothers with negative attitudes to nutrition advice tend to have a higher BMI might represent an interesting addition to the literature on factors influencing eating behaviors (as the BMI is one direct outcome of dietary choices). Although the direction of causality must be determined (for example, individuals with a higher BMI may tend to have negative attitudes to nutrition advice), it might be interesting to further investigate whether undesired nutrition advice from health experts might influence eating behaviors negatively. Above all, the 4-item scale “Attitudes towards nutrition advice” might be a useful tool for practitioners to evaluate the patient/client receptiveness of nutrition counseling. It showed a high reliability and good factor structure (see Table 2) and, to the authors’ knowledge, it is the first proposed scale to measure this specific aspect. Identification of individuals who are less open to nutrition advice is important in order to adapt the consultation/intervention and to avoid transgressive attitudes. It has been reported that individuals who are required to change their attitude to a high degree (e.g., changes in the diet) might act in the opposite direction of what is advocated by health professionals (transgressive attitude) (O’Keefe, 2015). Previous research found that parents feel overwhelmed with contemporary nutrition messages, rejecting and even repudiating healthy eating discourses (Moura & Aschemann-Witzel, 2021). In particular, Danish parents said that they prioritize having a sensible relationship with food rather than strictly complying with public nutrition recommendations (Gram & Grønhøj, 2015). Parents with this pattern might need an approach that differs from the classical nutrition one, focusing more on gastronomy and hands-on health solutions rather than on nutrition concepts (Poulain, 2009).

### Strengths and Limitations

This study represents an effort to optimize nutrition treatment for women in their roles as mothers of young children. An important strength of the proposed short-version scale is that it can be completed quickly, which is of special importance to professionals working in public health facilities. The scales have been further translated into other languages (Spanish and French) and successfully applied to a sample of 1500 women to identify segments of mothers in relation to barriers to healthy eating (Moura & Aschemann-Witzel, 2022). However, the results should be interpreted in light of their limitations, which include a specific population of mothers and self-reported measurements (BMI, healthiness of eating patterns). Further research is needed to validate fully the scales with other larger and more diverse samples. Because the questionnaire was developed in use of a sample of mostly well-educated Danish women, most of them with a slightly elevated BMI (mean BMI: 26), additional research is needed to determine the extent to which this instrument is appropriate for diverse populations differing in ethnicity, gender, demographics, and BMI. Furthermore, objective outcome measures (healthy eating objective indicators, as opposed to perceived healthiness) are needed to determine whether the scales can predict (un) healthy eating patterns, thus confirming the validation of the items to predict women’s health outcomes. The application of the questionnaire in a clinical setting would also determine its feasibility and applicability for use by health professionals.

## Conclusions for Practice

The “Social-cognitive measures of maternal barriers to healthy eating” questionnaire provided a potentially reliable theory-based tool for assessing constraints to healthy eating among mothers of young children living in Denmark. Exploratory and confirmatory factor analyses showed adequate/acceptable factorial validity and model fit, adequate internal consistencies, and relations with certain outcomes (perceived healthy eating and self-reported BMI).

Further testing of the questionnaire is needed, but these promising findings of reliability (Cronbach’s alpha values > 0.7 and even > 0.8 for some constructs) and initial validity (associations of the scales with perceived healthy eating and self-reported BMI) suggest that researchers and practitioners interested in identifying women who face difficulties while providing healthy foods and meals for themselves and their families may find the scales useful.

The questionnaire can be completed quickly, which is an important consideration when recruiting mothers of young children, who most likely have a busy routine. The questionnaire can also be self-administered (in the case of individuals with at least medium educational levels) and be filled in prior to consultations or recruitment, thus optimizing time. Moreover, as opposed to qualitative techniques, these quantitative scales provide a cost-effective method to collect data from large samples of women. These aspects can be important for professionals and researchers working with limited resources, which is often the case for professionals working with maternal and child health (Lucas et al., 2014).

Finally, although the questionnaire has not been evaluated in a clinical context, health practitioners may wish to incorporate items from it into their assessment strategies to facilitate nutrition counseling. Mostly, the use of the questionnaire by health professionals working with diverse communities would add important insights about its applicability in different settings.

### Electronic supplementary material

Below is the link to the electronic supplementary material.

Supplementary file1 (DOCX 273 KB)

## Data Availability

Some of the data generated during this study are included in this published article and its supplementary information file. Further datasets generated during and/or analyzed during the current study are available from the corresponding author upon reasonable request.

## Appendix 1

Social-cognitive measures of maternal barriers to healthy eating – version for health professionals.

**Table:**

*Part I: General barriers to healthy eating*	Score*				
(1) I do not have enough money to eat healthily					
(2) Most fresh fruit and vegetables do not look appealing in the store					
(3) Preferably, I spend as little time as possible cooking					
(4) I lack cooking skills to prepare healthy food					
(5) What we are going to have for dinner is very often a last-minute decision					
(6) At the end of the day I just don't have the energy to whip up a healthy meal					
(7) My family wastes too much food when I serve fruit and vegetables					
(8) Healthy meals are boring					
*Part II. Attitudes, capabilities, knowledge*					
(9) Compared to most other people, I know less about healthy eating					
(10) I am fed up with all the controversy about healthy eating among experts					
*(11) If I am in a challenging situation, I tend to find a way out*					
*(12) It’s important to me that the food I eat…*					
*(a) …helps to prevent diseases*					
*(b) …is good for my appearance (e.g. skin, hair, nails, teeth)*					

*Scores varying in a scale of 1-5: 1) Strongly disagree, 2) Disagree, 3) Neither agree nor disagree, 4) Agree, 5) Strongly Agree

*Reverse code: questions 11, 12a, 12b*

*Part I*: Higher values mean higher barriers to healthy eating.

Min: 8 Max:40.

*Part II*: Higher values mean more negative attitudes, and fewer capabilities and knowledge about healthy eating.

Min: 7 Max: 35.

## Appendix 2

Social-cognitive measures of maternal barriers to healthy eating—guidance for health professionals.

**Table:**

	Suggestions for guidance in case of high scores	Suggestions of intervention
*Part I: General barriers to healthy eating*		
(1) I do not have enough money to eat healthily	Decrease meat consumption and increase consumption of beans^a^	Culinary nutrition intervention with unusual parts of fruits and vegetables
	Buy frozen fruits and vegetables^a^	
	Cook at home (instead of eating out)	
	Find recipes with non-usual parts of fruits and vegetables	
(2) Most fresh fruit and vegetables do not look appealing in the store	Consider buying frozen fruits and vegetables	Classes on home gardening
	When you find good fruits and vegetables, consider buying bigger quantities to store in the freezer	
	If possible, consider growing some foods at home (e.g. herbs and spices)	
(3) Preferably, I spend as little time as possible cooking	Try to organize the kitchen before start cooking	Culinary nutrition intervention for women's needs
	Look for recipes that fit your needs (in terms of time, preferences)	
(4) I lack cooking skills to prepare healthy food	If possible, look for cooking blogs and channels that fit your needs (in terms of time for cooking, preferences)	Culinary nutrition and home economics intervention
(5) What we are going to have for dinner is very often a last-minute decision	Try to plan on the weekend what you are going to have the next week	
	Make a shopping list and try to stick to it	
(6) At the end of the day I just don't have the energy to whip up a healthy meal	Try to organize in the weekend the meals you are going to have during the week	Culinary nutrition intervention with tools to optimize cooking time
	Cook big quantities of food to keep in the freezer. You can use frozen leftovers on busy and tiring days	
	If it is within your possibilities, consider meal boxes services that provide ingredients for healthy meals	
(7) My family wastes too much food when I serve fruit and vegetables	Keep fruits and vegetables visible in the kitchen (to get children acquainted with them)	Culinary nutrition intervention with the whole family
	Keep small portions of vegetables in the freezer for yourself (if it’s not possible to get the children to eat them)	
(8) Healthy meals are boring	If possible, look for cooking blogs and channels that fit your preferences	Culinary nutrition intervention
*Part II. Attitudes, capabilities, knowledge*		
(9) Compared to most other people, I know less about healthy eating	Practitioners should be open to answering questions that are important for each patient/client	Nutrition classes
(10) I am fed up with all the controversy about healthy eating among experts	Practitioners should be open to answering questions that are important for each patient/client	Cooking classes with a focus on gastronomy (low focus on nutrition concepts)
	Hands-on advice on planning and cooking (as opposed to nutrition concepts) might be useful	
(11) If I am in a challenging situation, I tend to find a way out	–	–
(12) It’s important to me that the food I eat…	In case of a negative answer, practitioners should be open to answering questions that are important for each patient/client	Cooking classes with a focus on gastronomy (low focus on nutrition concepts)
(a) …helps to prevent diseases	Hands-on advice on planning and cooking (as opposed to nutrition concepts) might be useful	
(b) …is good for my appearance (e.g. skin, hair, nails, teeth)	In positive cases, it can be an opportunity to increase motivation for healthy eating	–

^a^Inspired by Widen and Siega-Riz (2010)
