# Supplementary material for: Exploring Barriers to Healthy Eating Among Women in Their Role as New Mothers with a Theory-Driven Questionnaire
Source: Matern Child Health J. 2023 Apr 3;27(7):1176–90. doi: 10.1007/s10995-023-03622-7 (PMC10232640; doi:10.1007/s10995-023-03622-7)
Supplement: Supplementary file 1 — Supplementary file1 (DOCX 273 KB) [file 10995_2023_3622_MOESM1_ESM.docx]

**Exploring barriers to healthy eating among women in their role as new mothers with a theory-driven questionnaire**

**Maternal and Child Health Journal**

Andreia Ferreira Moura^1,2*^- PhD

Jessica Aschemann-Witzel^2^- Prof. Dr. habil.

1.University of Nottingham, Department of Food, Nutrition and Dietetics. School of Biosciences, Sutton Bonington, Loughborough LE12 5RD, UK.

2.MAPP Centre for Research on Value Creating in the Food Sector, Department of Management, BSS, Aarhus University, Fuglesangsalle 4, 8210 Aarhus V, Denmark

*Corresponding author.

Andreia Moura: [andreia.moura@nottingham.ac.uk](mailto:andreia.moura@nottingham.ac.uk) Telephone number: +44 115 951 6121

Jessica Aschemann-Witzel: [jeaw@mgmt.au.dk](mailto:jeaw@mgmt.au.dk) Telephone number: +45 9350 8332

**Supplementary material: Methodological and statistical validation details**

**A. Questionnaire development and translation process**

The questionnaire was developed in English by the authors, who held several meetings to review the items and the scales. Three other experts (in families’ and consumers’ eating behaviors) were consulted to assess the appropriateness of items, and a language expert (in English) reviewed the grammar, syntax and organization of the items. The English version was then pre-tested through face-to-face interviews with 10 mothers. The mothers identified items that they could not answer, that were unclear, that had multiple meanings, or that were too complex. Based on the results of the interviews, the items were revised, and two items were removed due to low face validity.

The selected scales went through a translation process and cultural adaptation: the translation from English to Danish by a professional translator was followed by a back-translation; a revision by three researchers with experience in the development of eating behavior scales for Danish consumers, and a pre-test with five Danish mothers.

The complete version of the questionnaire and the references for each item are presented below.

**Social-Cognitive Measures of Maternal Barriers to Healthy Eating** **–references item by item.**

Part 1: General barriers to healthy eating

| 1.a. Personal, behavioral and environmental barriers to healthy eating  (Scores from 1- strongly disagree to 7- strongly agree) |
| --- |
| **Planning meals** |
| 1.I often ‘‘go with the flow’’ and do not plan meals  Inspired by Storfer- Isser & Musher-Eizenman, 2013 |
| 2.What we are going to have for dinner is very often a last-minute decision  Brunsø & Grunert, 1995 |
| 3.Usually I do not decide what to buy until I am in the shop.  Brunsø & Grunert, 1995 |
| **Purchasing** |
| **Price** |
| 4.The prices of healthy foods is too high  Developed |
| 5.I do not have enough money to buy fruit and/or vegetables  Developed |
| 6.I do not have enough money to eat healthily  Inspired by Storfer- Isser & Musher-Eizenman, 2013 |
| **Availability** |
| 7.It’s difficult to find fresh fruit and vegetables where I live  Developed |
| 8.Most fresh fruit and vegetables do not look appealing in the store  Cullen et al., 2000 |
| 9. I live far from supermarkets or vegetable shops with fresh foods  Developed |
| **Family** |
| 10.When my partner does the grocery shopping s/he often buys unhealthy foods that I wouldn’t buy  Developed |
| 11. My kids often add products to our grocery basket that I wouldn’t buy (if I shopped alone)  Developed |
| 12.It’s difficult not to buy the unhealthy foods my kids see in advertisements  Inspired by Haerens et al., 2009 |
| **Cooking** |
| **Interest/enjoyment in cooking (general)** |
| 13.I don't like spending too much time cooking  Brunsø & Grunert, 1995 |
| 14.Preferably, I spend as little time as possible cooking  Inspired by Candel, 2001 |
| 15.Cooking is a task that is best over and done with  Brunsø & Grunert, 1995 |
| **Cooking healthy foods** |
| 16.It is difficult to find ways to prepare vegetables  Inspired by Machin et al., 2018 |
| 17.I often lack inspiration to cook healthy dishes  Developed |
| 18.I lack cooking skills to prepare healthy food  Inspired by Bava, Jaeger & Park, 2008 |
| **Time constraints/ situational** |
| 19.I don’t have enough time to prepare healthy food  Developed |
| 20. On busy nights, our family’s main meal includes canned or frozen entrees, boxed mixes or microwaveable dinners.  Inspired by Horning, Fulkerson, Friend & Story, 2017 |
| 21.I often have to abandon cooking plans because of unexpected events (e.g. work demands, kid’s sickness or mood swings)  Inspired by Jabs et al., 2007 |
| 22.At the end of the day I just don't have the energy to whip up a healthy meal  Inspired by Fulkerson et al., 2011 |
| **Eating** |
| **Attitudes to healthy eating** |
| 23. Healthy foods don't taste as good as unhealthy foods  Inspired by Machin et al., 2018 |
| 24.Healthy foods taste bad  Inspired by Machin et al., 2018 |
| 25.Healthy meals are boring  Developed |
| 26.I wouldn’t try new healthy foods that I am not used to  Developed |
| **Family** |
| 27.I find it difficult to balance nutrition concerns and my kid’s food preferences  Inspired by Horning, Fulkerson, Friend & Story, 2017 |
| 28.I get tired of putting effort into trying new dishes that my family refuse to eat  Developed |
| 29.My family wastes too much food when I serve fruit and vegetables  Cullen et al., 2000 |
| 30.If I were to add more vegetables to my usual dishes, no one in my family would eat them  Cullen et al., 2000 |
| 31.If I were to serve fruit for dessert, no one in my family would eat it  Cullen et al., 2000 |
| **Social /situational aspects** |
| 32.I often find myself in situations where eating a lot of food seems to be expected (e.g., holidays, social occasions, work functions, business trips)  Developed |
| 33.There are constant offers of unhealthy snacks at my work/study place  Developed |
| 34.There are no healthy foods available at my work/study place  Developed |

*Notes*: “Developed” indicates that the item is based on knowledge gained through qualitative interviews, social media content analysis, literature review or several of these sources. “Inspired by” indicates that the phrasing of the statement is based on a specific study result with the reference given, while items directly taken from another published study and/or scale are indicated with the reference.

| 1.b. Attitudes to nutrition advice  (Scores from 1- strongly disagree to 7- strongly agree) |
| --- |
| 35.Nutrition advice from professionals is confusing  Inspired by O’Key & Hugh-Jones, 2010 and Moura & Aschemann-Witzel, 2021 |
| 36.I am fed up with all the controversy about healthy eating among experts  Inspired by Lupton & Chapman, 1995 and Moura & Aschemann-Witzel, 2021 |
| 37.I prefer to rely on my common sense for healthy eating, rather than listening to experts  Inspired by Lupton & Chapman, 1995 and Moura & Aschemann-Witzel, 2021 |
| 38.I don’t like to be told by health professionals what I should eat. I can decide on my own.  Inspired by Crossley, 2003 and Moura & Aschemann-Witzel, 2021 |

*Note*: “Inspired by” indicates that the phrasing of the statement is based on a specific study result with the reference given, while items directly taken from another published study and/or scale are indicated with the reference.

| 1.c Outcome expectations  (Scores from 1- Very true of me to 7- Very untrue of me) |
| --- |
| It’s important to me that the food I eat… |
| 39.Helps me to **prevent diseases**  Developed |
| 40.Is good for my **appearance** (e.g., skin, hair, nails, teeth)  Naughton, McCarthy & Mc Carthy, 2015 |
| 41.Helps me to **live longer**  Developed |
| 42.Helps me to **control my weight**  Naughton, McCarthy & Mc Carthy, 2015 |
| 43.Does not compromise **the environment**  Developed |

*Notes*: “Developed” indicates that the item is based on knowledge gained through qualitative interviews, social media content analysis, literature review or several of these sources. Items directly taken from another published study and/or scale are indicated with the reference.

**Part 2: Pre-established scales: Social cognitive measures**

| 2.a. General self-efficacy  Schwarzer, 1993  (Scores from 1- strongly disagree to 7- strongly agree) |
| --- |
| 1.Thanks to my resourcefulness (my capabilities), I know how to handle unforeseen situations |
| 2.I am usually able to deal efficiently with any kind of unexpected events |
| 3.If I am in a challenging situation, I tend to find a way out |
| 4.When I am confronted with a problem, I can usually find several solutions |
| 2. b. Subjective Knowledge  Flynn & Goldsmith, 1999  (Scores from 1- strongly disagree to 7- strongly agree) |
| 5.I know pretty much about healthy eating |
| 6.I do not feel very knowledgeable about healthy eating |
| 7.Among my circle of friends, I’m one of the “experts” on healthy eating |
| 8.Compared to most other people, I know less about healthy eating |
| 9.When it comes to healthy eating, I really don’t know a lot |

**B. Data Analysis**

**Exploratory Factor analysis (EFA)** with oblimin rotation was conducted for Part I of the questionnaire (developed or adapted items). The following were examined to select the best fitting model: loading values, scree plot, eigen values (>1), and the interpretability of the factor solution*.* Items with a factor loading of less than 0.4 were removed from the questionnaire (DeVon et al., 2007).

The Kaiser-Meyer-Olkin (KMO) measure confirmed the sampling adequacy for the factor analysis, KMO = 0.861. The Barlett’s Test of Sphericity was significant (p < .001) and supported the factorability of the items.

**Confirmatory Factor Analysis (CFA)** was conducted to evaluate the measurement model fit of the scales, and to confirm the potential relations of the items with certain outcomes (perceived healthiness of eating along the phases of becoming a mother and the BMI). A good model fit was indicated by the following indices: ratio of chi-square to degrees of freedom (χ ^2^/DF) < 2.5 and Root Mean Square Error of Approximation (RMSEA) ≤0.08. In addition, scores ≥ 0.9 were considered acceptable for the following indices: Comparative Fit Index (CFI), Incremental Fit Index (IFI), Tucker Lewis Index (TLI) (Blunch, 2015; Hooper et al., 2008).

**C. Statistical Validation**

A **reliability analysis** of Part II of the questionnaire (pre-existing scales on social-cognitive measures) revealed Cronbach’s alpha values > .8, as presented in the table below.

Table. Reliability Values of Pre-existing Scales

|  | Mean^a^ (SD) | Cronbach’s alpha |
| --- | --- | --- |
| ***General self-efficacy*** |  |  |
| 1. Thanks to my resourcefulness (my capabilities), I know how to handle unforeseen situations | 5.42 (1.12) | .891 |
| 2. I am usually able to deal efficiently with any kind of unexpected events | 5.27 (1.09) |  |
| 3. If I am in a challenging situation, I tend to find a way out | 5.61 ( .99) |  |
| 4. When I am confronted with a problem, I can usually find several solutions | 5.43 (1.07) |  |
| ***Subjective Knowledge*** | 2.82^b^ (1.12) | .829 |
| 1. I know pretty much about healthy eating^b^ |  |  |
| 2. I do not feel very knowledgeable about healthy eating | 2.99 (1.36) |  |
| 3. Among my circle of friends, I’m one of the “experts” on healthy eating^b^ | 4.19^b^ (1.51) |  |
| 4. Compared to most other people, I know less about healthy eating | 3.14 (1.24) |  |
| 5. When it comes to healthy eating, I really don’t know a lot | 2.93 (1.20) |  |

1. Values ranged from 1 (strongly disagree) to 7 (strongly agree), so higher scores mean higher self-efficacy
2. Reverse coded 1 (strongly agree) to 7 (strongly disagree) to indicate that higher scores mean lower subjective knowledge

**Confirmatory Factor Analysis (CFA)**. Figures 1 and 2 present a seven-factor model of 24 items correlating with potential outcomes (perceived healthiness of eating nowadays and BMI). For each of the scales, 3 items were included in the CFA, which the exception of factor 1: General barriers (6 items, out of 22, were included due to the length of this scale). The items were chosen based on the items that would affect the least the Cronbach’s alpha value if the item was deleted.

Although the Chi-square test was not significant for both outcomes (the perceived healthiness of eating nowadays and the BMI), other indices met the recommended criteria for an acceptable overall fit for both outcomes (e.g. for healthiness of eating nowadays RMSEA= .054; for BMI, RMSEA = .053). These findings confirm the measurement model. Thus, the suggested structural relations give an acceptable representation of the data. More specifically, standardized path coefficients show that general barriers accounted moderately for the variance in perceived healthiness of eating (beta value = - .39). The results thus suggest that the more barriers to healthy eating, the less healthy mothers perceive to eat compared to before motherhood (negative correlation). The low correlation values of some constructs (e.g. planning meals, attitudes to nutrition advice, outcome expectations, subjective knowledge) may partly be explained by the subjective nature of the outcome variable (*perceived* healthiness of eating), which is also a subjective comparison between eating habits nowadays and eating habits before being a mother (as opposed to the actual healthiness of the current diet).

The results considering the BMI as an outcome (a more objective measure) indicated various variables of influence. Standardized path coefficients show that three constructs (general self-efficacy, attitudes to nutrition advice, outcome expectations and subjective knowledge) accounted for the explained variance in BMI. Specifically, the lower the self-efficacy (beta = .44), the more negative the attitudes are toward nutrition advice (beta = 1.56), the fewer outcome expectations (beta = .51) and the lower scores in subjective knowledge (beta = .28), the more tendency to a higher BMI.


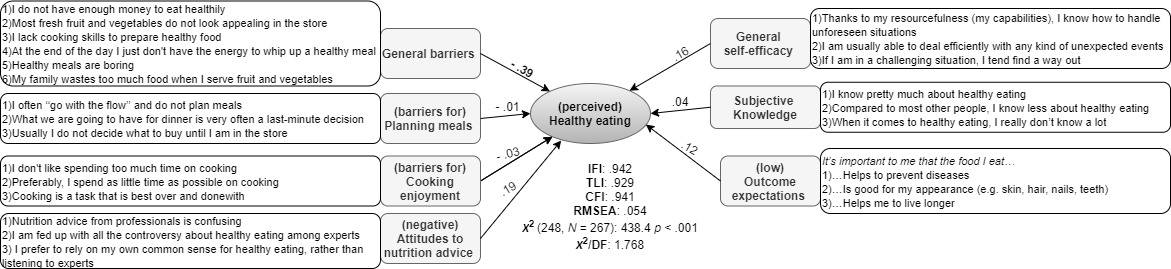


Figure 1. Results from confirmatory factor analysis correlating questionnaire constructs with perceived healthiness of eating nowadays (compared to before being a mother).

*Note*: IFI: Incremental Fit Index, TLI: Tucker Lewis Index, CFI: Comparative Fit Index, RMSEA: Root Mean Square Error of Approximation


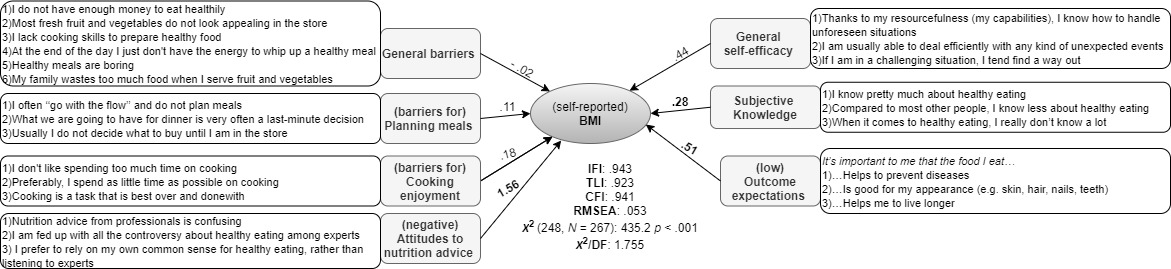


Figure 2. Results from confirmatory factor analysis correlating questionnaire constructs with BMI (self-report height and weight). *Note*: IFI: Incremental Fit Index, TLI: Tucker Lewis Index, CFI: Comparative Fit Index, RMSEA: Root Mean Square Error of Approximation

**References**

Bava, C. M., Jaeger, S. R., & Park, J. (2008). Constraints upon food provisioning practices in ‘busy’women's lives: Trade-offs which demand convenience. *Appetite, 50*(2-3), 486-498. doi:10.1016/j.appet.2007.10.005

Blunch, N. J. (2015). *Introduction to Structural Equation Modeling Using IBM SPSS Statistics and EQS* (2 ed.). UK: Sage.

Brunsø, K., & Grunert, K. G. (1995). Development and Testing of a Cross-Culturally Valid Instrument: Food-Related Life Style. *Advances in Consumer Research, 22*(1), 475-480.

Candel, M. J. (2001). Consumers' convenience orientation towards meal preparation: conceptualization and measurement. *Appetite, 36*(1), 15-28. doi:<https://doi.org/10.1006/appe.2000.0364>

Cullen, K. W., Baranowski, T., Rittenberry, L., Cosart, C., Owens, E., Hebert, D., & de Moor, C. (2000). Socioenvironmental influences on children's fruit, juice and vegetable consumption as reported by parents: reliability and validity of measures. *Public Health Nutrition, 3*(3), 345-356. doi:<https://doi.org/10.1017/S1368980000000392>

DeVon, H. A., Block, M. E., Moyle‐Wright, P., Ernst, D. M., Hayden, S. J., Lazzara, D. J., . . . Kostas‐Polston, E. (2007). A psychometric toolbox for testing validity and reliability. *Journal of Nursing Scholarship, 39*(2), 155-164. doi:10.1111/j.1547-5069.2007.00161.x

Fulkerson, J. A., Kubik, M. Y., Rydell, S., Boutelle, K. N., Garwick, A., Story, M., . . . Dudovitz, B. (2011). Focus groups with working parents of school-aged children: what's needed to improve family meals? *Journal of nutrition education and behavior, 43*(3), 189-193. doi:<https://doi.org/10.1016/j.jneb.2010.03.006>

Haerens, L., De Bourdeaudhuij, I., Barba, G., Eiben, G., Fernandez, J., Hebestreit, A., . . . Shiakou, M. (2009). Developing the IDEFICS community-based intervention program to enhance eating behaviors in 2-to 8-year-old children: findings from focus groups with children and parents. *Health Education Research, 24*(3), 381-393. doi:<https://doi.org/10.1093/her/cyn033>

Hooper, D., Coughlan, J., & Mullen, M. (2008). Structural equation modelling: guidelines for determining model fit. *Electronic Journal of Business Research Methods*(6), 53-60. doi:10.1037/1082-989X.12.1.58

Horning, M. L., Fulkerson, J. A., Friend, S. E., & Story, M. (2017). Reasons Parents Buy Prepackaged, Processed Meals: It Is More Complicated Than “I Don't Have Time”. *Journal of nutrition education and behavior, 49*(1), 60-66. e61. doi:<https://doi.org/10.1016/j.jneb.2016.08.012>

Jabs, J., Devine, C. M., Bisogni, C. A., Farrell, T. J., Jastran, M., & Wethington, E. (2007). Trying to find the quickest way: employed mothers’ constructions of time for food. *Journal of nutrition education and behavior, 39*(1), 18-25.

Machin, L., Aschemann-Witzel, J., Patino, A., Moratorio, X., Bandeira, E., Curutchet, M. R., . . . Ares, G. (2018). Barriers and Facilitators to Implementing the Uruguayan Dietary Guidelines in Everyday Life: A Citizen Perspective. *Health Education & Behavior, 45*(4), 511-523. doi:10.1177/1090198117744243

Moura, A., & Aschemann-Witzel, J. (2021). Perspectives on sugar consumption expressed on social media by French-speaking and Danish-speaking parents. *Social Science & Medicine, 270*, 113636. doi:10.1016/j.socscimed.2020.113636

Naughton, P., McCarthy, S. N., & McCarthy, M. B. (2015). The creation of a healthy eating motivation score and its association with food choice and physical activity in a cross sectional sample of Irish adults. *International Journal of Behavioral Nutrition and Physical Activity, 12*(1), 74. doi:<https://doi.org/10.1186/s12966-015-0234-0>

Schwarzer, R. (1993). *Measurement of perceived self-efficacy: Psychometric scales for cross-cultural research*. Berlin: Freie Universität Berlin, Instituit fúr Psychologie.

Storfer-Isser, A., & Musher-Eizenman, D. (2013). Measuring parent time scarcity and fatigue as barriers to meal planning and preparation: quantitative scale development. *Journal of nutrition education and behavior, 45*(2), 176-182. doi:<https://doi.org/10.1016/j.jneb.2012.08.007>
